# Home-based Pulmonary Rehabilitation in COPD: Bridging Evidence and Practice for Comprehensive Patient-centered Care

**DOI:** 10.5041/RMMJ.10560

**Published:** 2025-10-31

**Authors:** Anchal Thakur, Kanika Bhatia

**Affiliations:** Department of Physiotherapy, Maharishi Markandeshwar Institute of Physiotherapy and Rehabilitation, Maharishi Markandeshwar (Deemed to be University), Mullana-Ambala, Haryana, India

**Keywords:** Chronic obstructive pulmonary disease (COPD), home-based rehabilitation, patient-centered care, pulmonary rehabilitation

**To the Editor**,

We read with great interest the article by Kasim et al., “From Breathlessness to Better Living: Transforming COPD Care with Home-based Pulmonary Rehabilitation,” recently published in *Rambam Maimonides Medical Journal*. We would like to congratulate the authors on their important and timely contribution to the topic of pulmonary rehabilitation (PR) in chronic obstructive pulmonary disease (COPD).^1^ Their study focused on the effects of an organized 12-week home-based PR program in improving pulmonary function and decreasing disability, with particular emphasis on activities of daily living and participation, using the World Health Organization’s Disability Assessment Schedule 2.0 (WHODAS 2.0) outcome measures. Their findings are important as they emphasize the value of PR as a complement, and even an alternative, to pharmacological management of the patient’s symptoms, adding a broader patient-centered rehabilitation model to COPD care.

Chronic obstructive pulmonary disease is still one of the leading causes of mortality and morbidity worldwide, with more than 3 million deaths annually.^2^ The disease markedly diminishes the quality of life of patients suffering from chronic dyspnea, physical deconditioning, discrete exacerbations, and social isolation.^3^ While medications such as bronchodilators and inhaled corticosteroids are still the primary therapeutic options, they have limited efficacy in addressing the multidimensional burden that patients with COPD experience in daily living.^4^ Hence, the importance of PR is consistently endorsed by international guidelines as a foundation of comprehensive COPD management.^5^ Despite the established benefits of PR, relatively few patients enroll in conventional center-based programs, partly due to barriers such as distance, transportation, cost, and limited availability, especially in under-resourced settings.^6^ Kasim et al.’s findings are therefore timely, with good evidence that home-based PR may fill this gap in access and achieve clinically meaningful improvements.

An important strength of the study was the use of WHODAS 2.0, which allowed for a multidimensional measure of disability rather than just a measure of pulmonary function using spirometry. Improvements in pulmonary function, like force vital capacity (FVC) and forced expiratory volume in 1 second (FEV1), are important from a clinical standpoint; the wider message of PR, however, is the ability to fundamentally improve participation in daily life activities and social roles.^1^ Kasim et al. demonstrated improvements in the life activities and participation domains of WHODAS 2.0, indicating that the nature of rehabilitation is holistic; in other words, PR addresses both physiological and psychosocial facets of COPD. Their results are consistent with earlier studies that have validated WHODAS as an appropriate measure of disability in COPD, allowing clinicians to have a more patient-centered understanding of outcomes.^7^,^8^

In addition, Kasim et al. identified areas for consideration. The absence of a comparison group curtails the strength of causal inference, as observed changes may be due to natural variation in the disease, enhanced motivation, or the Hawthorne effect, etc. Because of this, randomized controlled trials are always necessary to determine efficacy and verify that external factors do not affect findings.^9^ In addition, self-report of adherence to the program was assessed during monthly phone calls and may have overestimated adherence, although this was convenient. In future studies, the use of tele-rehabilitation tools, such as wearable sensors, video conferencing, and mobile applications could allow for more objecttive adherence measures, improve real-time feedback for patients, and, possibly, enhance engagement and outcomes.^10^

While Kasim et al. reported improvements in life activity and participation, other important domains, such as mobility and cognition, did not demonstrate comparable improvements.^1^ This is consistent with emerging evidence showing that COPD is commonly associated with cognitive impairment due to chronic hypoxemia and systemic inflammation. Cognitive deficits may limit self-management and compliance, directly influencing outcomes. To improve cognition, rehabilitation programs can incorporate cognitive stimulation and dual-task training.^9^ The mobility impairment seen in individuals with COPD is commonly the result of muscle weakness, balance issues, and sarcopenia, which are generally not addressed with endurance-based training in physiotherapy. To improve mobility outcomes, resistive training, gait training, and balance training should all be part of any rehabilitation program. This underscores the need for multidimensional approaches in rehabilitation, including nutritional counseling, psychological support, and social reinforcement; combined they will ensure that rehabilitation addresses the major underlying determinants of disability.

The implications of Kasim et al.’s study are particularly relevant in high-burden COPD countries such as India, where rehabilitation resources may be lacking.^10^ In these settings, PR delivered in the home by physiotherapists, nurses, or community health workers is often less costly or more realistic from a scaling perspective. Involving family members in the process, which the authors discussed, could enhance patient adherence and support. Further, if home-based PR decreases future hospitalizations and exacerbations, this may reduce the overall economic burden associated with COPD, which remains a major hurdle for low- and middle-income countries’ health systems. For these reasons, the inclusion of home-based PR in national respiratory care programs and covering the expenses through public and private insurance may be warranted. Preparing healthcare providers to conduct the structured home-based PR protocols will be essential for success.

Looking ahead, further investigation should aim to confirm the sustainability of home-based PR benefits in the long term; it remains unclear whether improvements measured at 12 weeks can be sustained in the longer term. Further studies should also investigate the use of tele-rehabilitation and digital monitoring platforms that may further extend access for remote populations and support patient adherence. In addition, personalizing the intervention for individual patients based on overall disease severity and management of comorbidities and psychosocial profiles is likely to optimize overall benefit and outcomes. Personalization can ensure patients receive oxygen-assisted training if they have advanced disease, and patients with depression or other cognitive impairments can receive appropriate psychosocial and cognitive-driven interventions. Equity issues affecting the accessibility of home-based PR, such as gender, socioeconomic status, and rural-–urban distinctions, should also be examined in future studies to ensure access to all patient populations.

In conclusion, the study by Kasim et al. provides important evidence in support of home-based PR as a viable, effective, and patient-centered approach to COPD care.^1^ By showing improvements in both pulmonary function and disability, the authors illustrate the value of rehabilitation services beyond just pharmacological therapy for restoring independence and social participation to patients. While additional controlled and long-term studies are needed, embedding home-based PR into the standard pathways of COPD care is an important step toward a sustainable and comprehensive management solution to this global health issue. We strongly support the authors’ work and hope that it stimulates clinicians, researchers, and policymakers to develop home-based PR as a core component of a holistic approach to COPD care.

## Abbreviations

COPD: chronic obstructive pulmonary disease
PR: pulmonary rehabilitation
WHODAS 2.0: World Health Organization’s Disability Assessment Schedule 2.0.
